# Anti-IL-5 biologics and rheumatoid arthritis: a single-centre 500 patient year exposure analysis

**DOI:** 10.1136/rmdopen-2023-003583

**Published:** 2023-12-19

**Authors:** Nathan J Dean, Ian J Clifton, Rashad Salman, Charles Bridgewood, Jacquie Nam, Tom Macleod, Dennis G McGonagle

**Affiliations:** 1 Rheumatology, Leeds Teaching Hospitals NHS Trust, Leeds, UK; 2 Respiratory Medicine, Leeds Teaching Hospitals NHS Trust, Leeds, UK; 3 Leeds Institute of Rheumatic and Musculoskeletal Medicine, University of Leeds, Chapel Allerton Hospital, Leeds, UK

**Keywords:** Arthritis, Arthritis, Rheumatoid, Biological Therapy, Immune System Diseases

## Abstract

**Objective:**

The increasing use of biological therapies has led to the paradoxical finding that monoclonal antibody therapy for one inflammatory disease can sometimes induce another inflammatory disease. Recently, the use of anti-IL-5 (IL, interleukin) antibody therapies for severe asthma has been associated with the onset of rheumatoid arthritis (RA) and other inflammatory rheumatological disease. We undertook this audit to identify the prevalence of this finding across a large clinical cohort of patients receiving anti-IL-5 therapy.

**Methods:**

All patients currently receiving mepolizumab or benralizumab for severe asthma across the Leeds Teaching Hospitals NHS Trust’s (LTHT) Respiratory Service were included. Electronic records for each patient were searched to identify clinical and biochemical manifestations of inflammatory rheumatological disease following the initiation of anti-IL-5 therapy.

**Results:**

142 patients, with a mean duration of 3.5 years on therapy, were included (89 mepolizumab, 53 benralizumab). 17 patients developed new arthralgias (nine mepolizumab, eight benralizumab), however only one of these patients (on mepolizumab) had raised acute phase reactants and newly positive anti-CCP antibody (ACPA) and rheumatoid factor and was the only patient to receive a formal diagnosis of RA.

**Conclusion:**

Although ACPA positive RA has now been reported in a handful of case reports, we noted a very low rate of evolution into RA or inflammatory arthritis, at least in the short-medium term under anti-IL-5 therapy. This challenges the emerging suggestion that anti-IL-5 biologics may be triggering RA.

**WHAT IS ALREADY KNOWN ON THIS TOPIC**

- Monoclonal therapy for one inflammatory disease may paradoxically trigger another inflammatory disease.
- Recent case reports have implicated an association between anti-IL-5 (IL, interleukin) antibody therapy used to treat severe asthma and the development of rheumatoid arthritis (RA).

**WHAT THIS STUDY ADDS**

- Out of 142 patients within our asthma service taking anti-IL-5 antibody therapy for at least 1 month, and with a mean duration of 3.5 years on therapy, only one developed RA suggesting that RA is a relatively uncommon complication in the short-medium term.

**HOW THIS STUDY MIGHT AFFECT RESEARCH, PRACTICE OR POLICY**

- Treating clinicians should be mindful of the possibility of developing inflammatory arthritis following the initiation of anti-IL-5 therapy and ensure appropriate review and assessment should their patient develop arthralgia as, while uncommon, these could represent a significant source of morbidity.

## Introduction

There has been a wide adoption of monoclonal antibody therapy in rheumatology, respiratory medicine, and an increasing number of specialties for the treatment of many inflammatory diseases. Of particular interest is that monoclonal therapy for one inflammatory disease may paradoxically trigger another inflammatory disease. Pertinent examples include tumour necrosis factor inhibitor therapy triggering multiple sclerosis,^1^ interleukin 17 (IL-17) therapy for psoriasis linked to inflammatory bowel disease^2^ and more recently IL-4/13 blockade used for atopic dermatitis being associated with de novo psoriasis and arthritis.^3 4^

Arthralgias are a known adverse effect of anti-IL-5 biologics,^5 6^ however, a few recent case reports have found this association may extend to inflammatory arthritis such as RA.^7 8^ The prevalence of these findings across a wider cohort of patients remains relatively unknown. Here we present an audit from a large, single-centre’s severe asthma service which looks for the prevalence of RA across all patients being treated with mepolizumab and benralizumab, two commonly used anti-IL-5 therapies.

## Methods

All patients with severe eosinophilic asthma across the Leeds Teaching Hospitals NHS Trust’s (LTHT) Respiratory Service, who had received at least 1 month of mepolizumab or benralizumab therapy, were included in this clinical audit.

Each patient’s electronic records, including hospital records, clinic letters, general practitioner (GP) records and electronic pathology results were searched. We recorded whether patients had presented with any signs or symptoms of synovitis (eg, joint pain, swelling and tenderness) either prior- or post-commencing biologics, whether their serology (rheumatoid factor (RF) and/or anti-CCP antibody (ACPA)) and acute phase reactants (C- Reactive Protein (CRP) and/or Erythrocyte Sedimentation Rate (ESR)) had been measured and the timing and duration of their symptoms. Using this information, we then calculated the number of points each patient with symptoms would score on the ACR/EULAR 2010 Rheumatoid Arthritis classification criteria.^9^ We also recorded the dose of routine steroids the patients were receiving prior to starting biologics, and whether they were weaned off steroids within 1 year of commencing biologics. Finally, we recorded whether the patients had been seen in our early arthritis clinic and received a formal diagnosis on an inflammatory arthritis.

## Results

A total of 142 patients (57 males, 85 females and mean age 58.2 years old) were being treated with anti-IL-5 biologics under the LTHT’s severe asthma clinic, with a mean duration of 3.5 years on therapy. Eighty-nine were on mepolizumab and 53 on benralizumab. The mean daily dose of steroids prior to starting anti-IL-5 therapy was 6.0 mg prednisolone, reducing to 3.1 mg at 1 year post-therapy. Seventy-five patients were steroid-free after 1 year of therapy.

Only one patient among 500 patient years of exposure to anti-IL-5 therapy received a formal diagnosis of RA suggesting an overall annual incidence of 20 cases per 10 000 patients (95% CI 2.8 to 142). This man in his 70s presented to our early arthritis clinic 18 months after having been started on mepolizumab for his severe eosinophilic asthma. Prior to starting biologics, his asthma had been poorly controlled with salbutamol, budesonide/formoterol combination inhaler, tiotropium inhalers and daily low-dose oral corticosteroids. Within weeks, he developed symmetrical arthralgia involving small joints, particularly his wrists and knuckles, as well as significant early morning stiffness lasting more than 1 hour. On examination, he had clinical synovitis in the wrists and metacarpophalangeal joints bilaterally, as well as right shoulder capsulitis with limiting range of motion.

**Figure 1:**
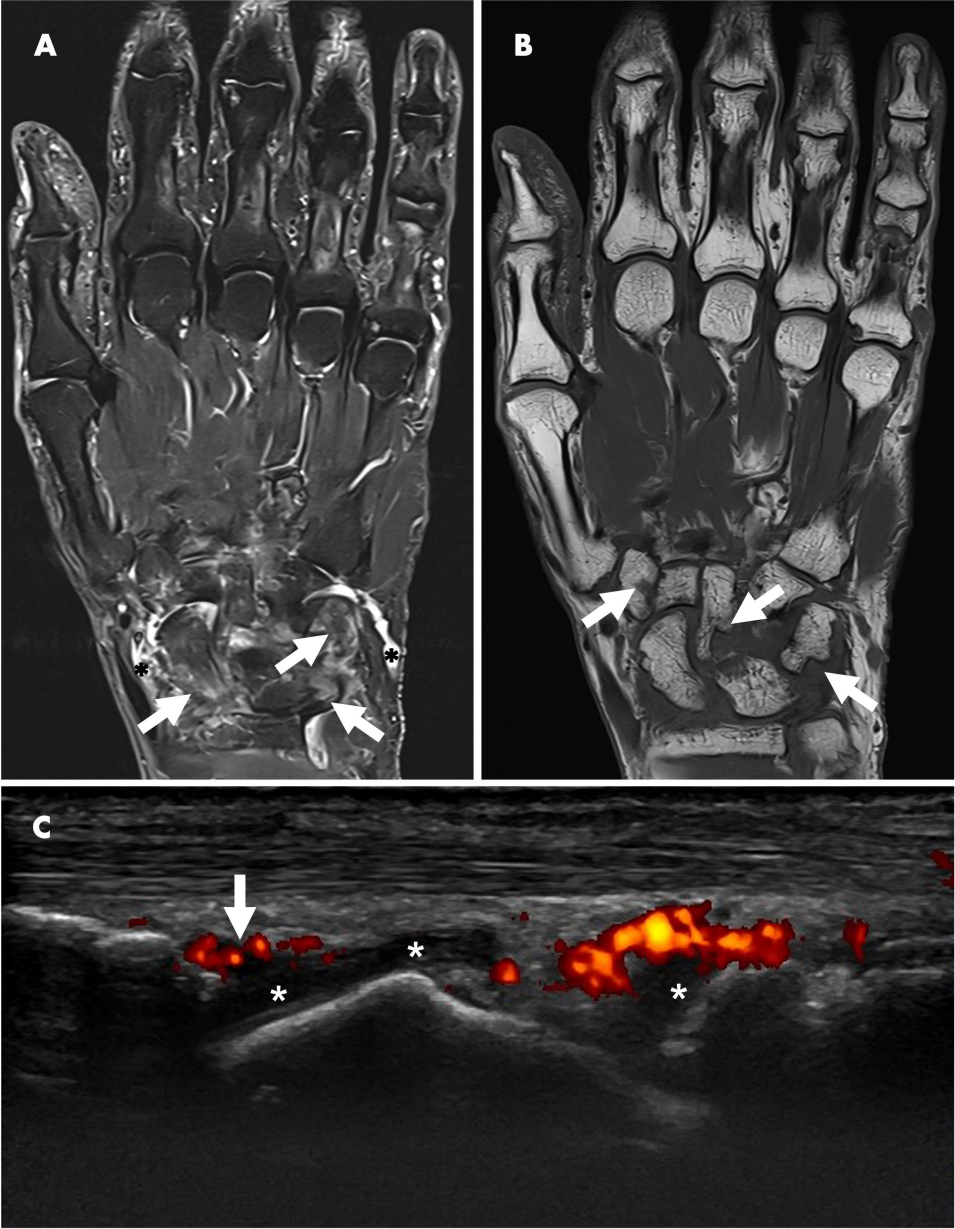
(A) Fat suppression MRI of the left wrist and MCPs showing extensive bone oedema (white arrows) and joint effusion (black asterisk). (B) T1-weighted MRI of the left wrist showing diffuse erosions of the left wrist (white arrows). (C) Longitudinal ultrasound image showing synovitis of the right wrist with grey scale (white asterisk) and power Doppler (white arrow).

Blood tests revealed a raised CRP of 24 mg/L, White Cell Count (WCC) 8.77 10 × 9 /L, RF of 263.2 iu/mL (normal<14.0) and an ACPA of >300 U/mL (normal<2.99). Ultrasound imaging of the hands and wrists showed bilateral grade II grey scale with grade II power Doppler (figure 1) with bilateral wrist erosions. There was hypoechogenicity of the left extensor carpi ulnaris tendon with some associated grey scale and power Doppler. MRI of the left hand revealed extensive subchondral bone marrow oedema (figure 1) and multiple erosions across all carpal bones and carpometacarpal joints (figure 1).

He was diagnosed with RA as per the American College of Rheumatology (ACR)/EULAR classification criteria and started on prednisolone 10 mg daily to control the inflammation, followed by sulfasalazine 1 month later as the disease modifying agent. He was followed-up in rheumatology clinic 2 months later and showed significant improvements: the joint pain and swelling had settled, and while he still experienced early morning stiffness, this was less debilitating. His inflammatory markers had also resolved with CRP<5.0 mg/L and WCC 9.31 10 × 9 /L.

Of the remaining 141 patients, 16 developed bilateral polyarthralgia of greater than 1 month duration (eight mepolizumab and eight benralizumab), with a median onset of 12 months after commencing a biological therapy. Of these patients 9/16 were tested for RF and ACPA and in all cases, their serology was negative; 15/16 patients had acute phase inflammatory markers measured and these were only elevated in three patients. All 16 of these patients were on a maintenance dose of prednisolone prior to starting the biologic (mean dose 10.1 mg/day), with 10 of them completely weaned off steroids within 12 months.

Using the information available from the patient’s electronic records, the mean number of points scored on the ACR/EULAR RA criteria was 3.2 (range 1–6). The patient who scored six points was reviewed in the early arthritis clinic and the symptoms were felt to be more in keeping with osteoarthritis than an inflammatory arthritis. Similarly, none of the other patients had received a confirmed diagnosis of inflammatory arthritis by either their GP or by a rheumatologist.

Only one other patient became newly RF positive (17.1 iu/mL), 1 month after commencing mepolizumab; however, this seemed to be an incidental finding as the patient had a broad set of bloods taken while admitted to the intensive care unit for a severe exacerbation of asthma, and at no point since has complained of rheumatological symptoms.

We were unable to access the GP records for 37 patients and as such could not review whether they had presented to their GPs with new rheumatological symptoms. However, we were able to access their pathology test records electronically and found no evidence of positive RA serology in any of these patients and no rheumatological referrals to our centre that has a well-developed early RA network.

## Discussion

There is an emerging interest in IL-5 blockade and the potential development of RA. We present a single-centre’s experience of 500 patient years on anti-IL-5 monoclonal antibody exposure therapy for severe asthma.

As expected, arthralgias were a relatively common side-effect of anti-IL-5 therapy. As for progression to RA, we found only one convincing case. While relatively low, the implied annual incidence of 20 cases per 10 000 patients is several fold higher than the annual incidence of RA in the UK (1.5 per 10 000 men and 3.6 per 10 000 women).^10^ Given the wide CIs, however, no firm conclusions can be offered in relationship to our single case and to the relative risk of RA following anti-IL-5 therapy.

A major confounding variable is the weaning of steroids in most patients started on biologics. This poses a challenge in associating the development of symptoms with the initiation of the anti-IL-5 therapy, as opposed to the withdrawal of steroids unmasking a pre-existing disease. Additionally, one must consider whether the risk of developing RA is modified by the underlying condition, and indeed there is some evidence to suggested that asthma may be positively associated with RA.^11^ However, these population-based studies look at asthma as a whole, rather than divided into its endotypes (eg, eosinophilic vs neutrophilic asthma) and as such these have not yet challenged the conventional belief that Th1 and Th2 diseases are inversely related.

Emerging evidence has implicated a core role for regulatory eosinophils (rEos) in the resolution of RA.^12^ In murine models of RA, the expansion of rEos in the synovial fluid as a by-product of inducing eosinophilic asthma was sufficient in bringing about remission of arthritis, and inhibiting the IL-5 pathway would subsequently induce relapse of the arthritis.^12^ Further evidence supporting a role for rEos in RA can be found at a genetic level where Eotaxin-3, one of the main drivers of eosinophil recruitment, has single nucleotide polymorphisms associated with RA^13^ and from studying the role of IL-5 in Th2 responses to Helminth infections,^14^ with mouse models of RA also identifying Helminth infections as protective.^15^ Hence, the suggestion that the expansion of eosinophils in the synovium ‘regulate’ the proinflammatory Th1 pathways driving synovial inflammation.^12^ This invites the notion that in a patient with subclinical, yet endogenously controlled, synovial inflammation, removing rEos by administering anti-IL-5 therapeutics may tip the balance in favour of inflammation and permit symptomatic disease. However, if there is little proinflammatory Th1 synovial activity in the first place, then inhibiting rEos with anti-IL-5 biologics may be insufficient to precipitate an inflammatory arthritis.

Interestingly, there is debate as to whether rEos are depleted to varying degrees depending on the anti-IL-5 biologic used. In mice, inflammatory eosinophils (iEos)—the primary targets of anti-IL-5 biologics in asthma—may be dependent on IL-5 for activity, whereas rEos may not be.^16^ This would suggest that benralizumab, a high-affinity IL-5 receptor antagonist,^17^ would deplete both iEos and rEos through NK-mediated killing, whereas mepolizumab, an anti-IL-5 monoclonal antibody,^17^ may deplete iEos but keep rEos intact. However, this idea has recently been challenged with evidence that anti-IL-5 treatment depletes all populations of eosinophils.^18^ Whether this distinction would result in a different pattern of adverse effects in patients remains unclear, notably as the patient who developed RA in this report was receiving mepolizumab.

As an audit, this study serves to identify the prevalence of a relatively rare complication of anti-IL-5 therapy. We were unable to find clear evidence for a pattern of emergent RA nor other inflammatory arthritis in our cohort of 142 patients. Further studies may be required to characterise the nature and significance of these findings in clinical groups and to identify whether there is an actual association between novel anti-IL-5 biologics and RA.

## Data Availability

The data that support the findings of this study are available upon reasonable request.
